# Hemothorax as the Initial Manifestation of *KRAS G12D* Positive Pulmonary Pleomorphic Carcinoma: A Case Report

**DOI:** 10.1002/rcr2.70454

**Published:** 2026-01-05

**Authors:** Takuma Ikeda, Hirotaka Matsumoto, Shigenari Iwagaki, Ryo Ogawa, Yushi Shimamura, Emiko Saito, Takehisa Fukada, Hiroaki Sakai

**Affiliations:** ^1^ Department of Respiratory Medicine Hyogo Prefectural Amagasaki General Medical Center Amagasaki‐shi Hyogo Japan; ^2^ Department of Thoracic Surgery Hyogo Prefectural Amagasaki General Medical Center Amagasaki‐shi Hyogo Japan

**Keywords:** hemothorax, KRAS G12D, non‐small‐cell lung cancer, pulmonary pleomorphic carcinoma

## Abstract

Pulmonary pleomorphic carcinoma (PPC) is a rare subtype of lung cancer that is characterised by rapid progression and poor prognosis. Hemothorax as the initial clinical presentation of PPC is exceptionally rare and, to the best of our knowledge, has not been previously reported. Here, we report a rare case of a KRAS G12D‐mutated PPC penetrating the visceral pleura, leading to rapid tumour growth and uncontrolled hemothorax. In addition to the high proliferative and invasive potential of the tumour, the oncological properties associated with the KRAS G12D mutation likely precipitated both the abrupt onset and recurrence of massive hemothorax. Early recognition, complete macroscopic resection to achieve definitive haemostasis, and prompt initiation of postoperative therapy are essential to improve clinical outcomes.

## Graphical abstract

We report a rare case of KRAS G12D positive pulmonary pleomorphic carcinoma penetrating the visceral pleura, leading to rapid tumour growth and uncontrolled hemothorax. In addition to the high proliferative and invasive potential of the tumour, the oncological properties of the KRAS G12D mutation likely precipitated both the abrupt onset and recurrence of massive hemothorax. Early recognition and complete macroscopic resection to achieve definitive haemostasis are essential to improve clinical outcomes.

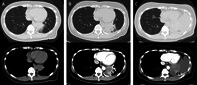

## 1 Introduction

Pulmonary pleomorphic carcinoma (PPC) is a rare sarcomatoid carcinoma, accounting for only 0.1%–0.3% of all lung cancers. It is defined histologically as a poorly differentiated carcinoma containing at least 10% spindle or giant cells. PPC progresses rapidly, and even in the early stages, it frequently invades the pleura and vasculature, resulting in a high risk of postoperative recurrence. In advanced stages, the prognosis is worse, with a reported median survival time of 2 months and a 5‐year overall survival of 2.2% [1]. Cavitation and pleural invasion are recognised as independent risk factors for mortality [2]. Hemothorax as the presenting feature of PPC is extraordinarily rare and has not, to our knowledge, been previously reported. Herein, we report the first case of massive hemothorax caused by a KRAS G12D‐mutated PPC.

## 2 Case Report

**FIGURE 1:**
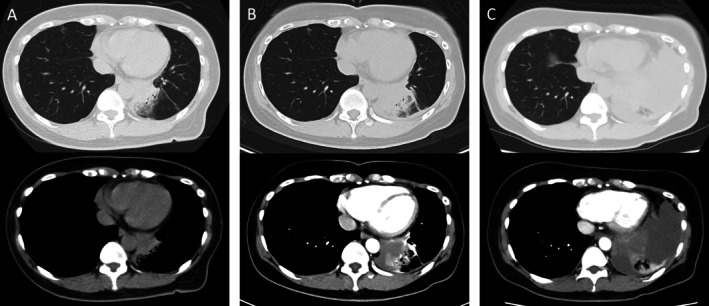
Computed tomography (CT) image. Initial CT (A) shows consolidation in the left lower lobe with surrounding ground‐glass opacity. Three days later, follow‐up CT (B) demonstrates an irregularly marginated nodule within the consolidation and a newly appeared small left pleural effusion. One week later, CT (C) shows marked enlargement of the irregular mass along with a large amount of high‐attenuation pleural effusion, consistent with hemothorax.

**FIGURE 2:**
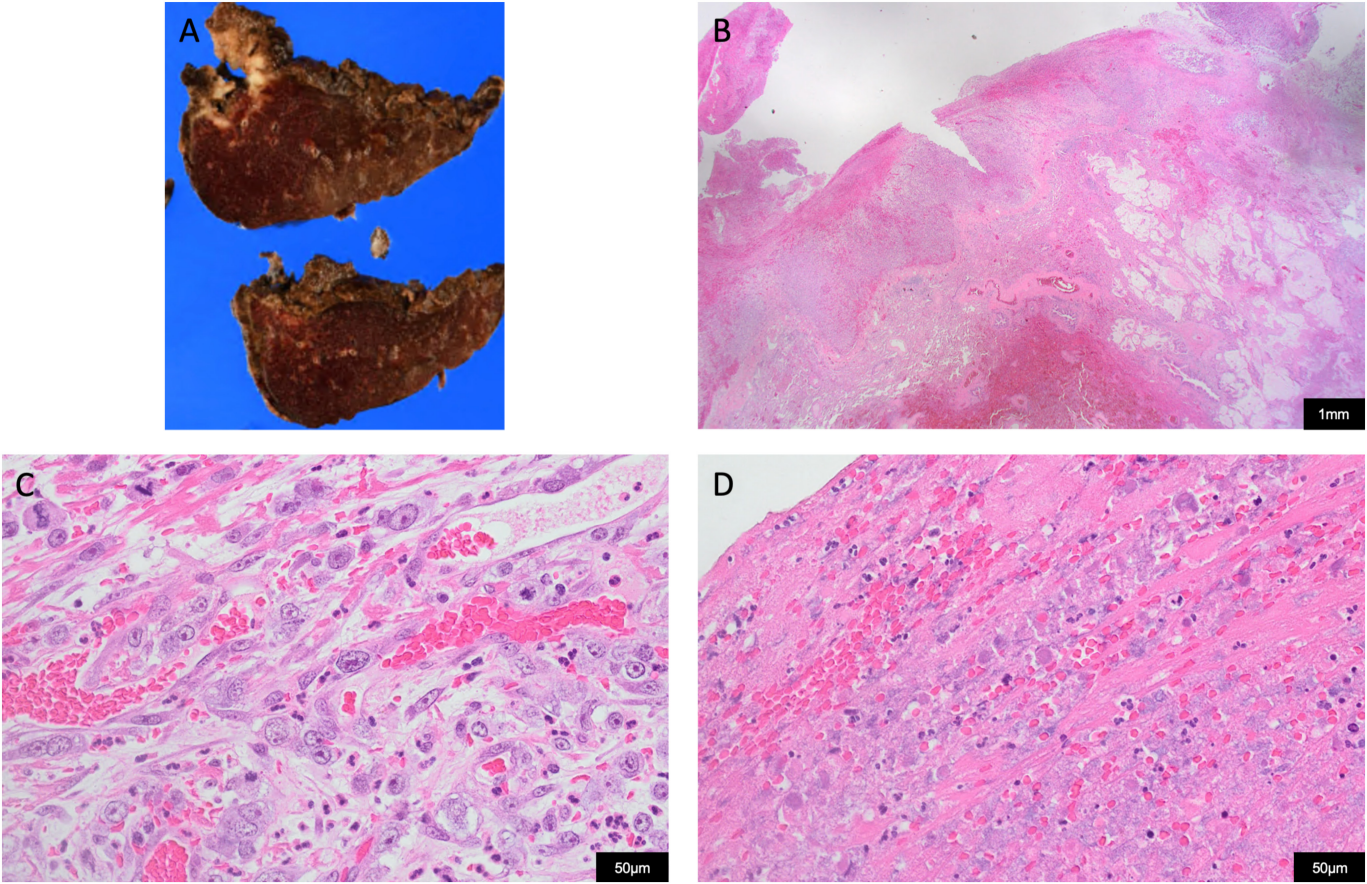
Pathological findings. (A) Resected specimen of the left lower lobe showing a 4.5 cm mass with central cavitation. (B) Lung, Haematoxylin and eosin (HE) staining (×12.5): The tumour infiltrates both the lung parenchyma and the visceral pleura, accompanied by haemorrhage. (C) Lung, HE staining (×400): The tumour is composed of a mixture of spindle cells and multinucleated giant cells, consistent with pleomorphic carcinoma. Numerous delicate vascular structures are observed. (D) Lung, HE staining (×400): Extensive necrosis and disruption of neovascularization are evident within the tumour.

A 56‐year‐old woman, a never‐smoker with no notable medical history, presented with a three‐day history of hemoptysis. Chest computed tomography (CT) revealed an infiltrative opacity surrounded by ground‐glass attenuation in the left lower lobe (Figure 1A). Community‐acquired pneumonia was suspected, and antibiotics were administered. Three days later, CT showed progression of the lesion and a small left pleural effusion (Figure 1B). One week later, the pleural effusion expanded abruptly, and thoracentesis yielded 1000 mL of bloody fluid, confirming a hemothorax. Contrast‐enhanced CT revealed a rapidly enlarging mass in the left lower lobe with partially hypoenhanced areas (Figure 1C). No active extravasation was observed, but bleeding was suspected. On the second day of hospitalisation, video‐assisted thoracoscopic left lower lobectomy was performed. A large volume of hemorrhagic pleural effusion was removed, and a 45‐mm tumour was found penetrating the visceral pleura with continuous oozing. To prioritise haemostasis, we performed an extensive resection of the tumour; however, only a palliative resection (R2) was achieved. Histopathology confirmed pulmonary pleomorphic carcinoma (pT2aN2bM1a, Stage IVA; PD‐L1 TPS: 30%; KRAS G12D mutation). Numerous necrotic foci and disrupted neovessels were observed on the visceral‐pleural surface (Figure 2).

On postoperative day 9, the patient experienced a second massive hemothorax. Although intrathoracic hematoma evacuation and electrocautery were performed, the rapid regrowth of the residual tumour with diffuse surface oozing made complete haemostasis impossible. Two subsequent pleurodeses failed to control the bleeding, and the patient died 30 days after symptom onset.

## 3 Discussion

Hemothorax is a rare complication of lung cancer and is exceedingly uncommon in PPC, with no reported cases to the best of our knowledge. In this case, the hemothorax led to the diagnosis of PPC. The primary lesion was surgically removed, yet the patient developed a second massive hemothorax postoperatively and ultimately died.

The immediate causes of bleeding were the rupture of visceral pleura overlying the necrotic tumour and the disruption of fragile neovessels. CT showed an irregular mass with hypoenhanced areas—a feature observed in 90% of PPCs that reflects intratumoral necrosis [2]. Histology confirmed extensive necrosis along with numerous fragile, disrupted neovessels. Therefore, we speculate that rapid tumour growth led to central ischemic necrosis, and once this necrotic focus breached the pleura, the simultaneous disruption of abundant fragile neovessels amplified the haemorrhage and resulted in an intractable hemothorax.

The presence of a KRAS G12D mutation may further exacerbate tumour progression and bleeding. KRAS‐mutated PPC is associated with poor prognosis [3]. KRAS G12D, a variant more commonly found in never‐smokers, activates the PI3K–AKT pathway, promoting cell proliferation and inhibiting apoptosis [4]. In particular, mTORC1 activation upregulates HIF‐1α expression and drives angiogenesis [5]. These molecular features likely contributed to the tumour's rapid growth, necrosis, and angiogenesis that precipitated hemothorax in our patient.

Early diagnosis and a complete surgical resection are critical for local control of PPC. Clinically, PPC are often misdiagnosed as pneumonia or atelectasis and can enlarge dramatically within a short period. Nonetheless, the combination of hemoptysis, an infiltrative lesion, and rapidly increasing pleural effusion should raise suspicion of malignant effusion, particularly aggressive PPC. When PPC is identified, a complete macroscopic resection should be pursued whenever feasible, which minimises the risk of rebleeding and ensures timely initiation of postoperative systemic therapy. In our case, priority was given to haemostasis, resulting in R2 resection. Massive rebleeding and tumour regrowth occurred only 9 days postoperatively, precluding postoperative treatment. Hence, early diagnosis and surgical strategies aimed at achieving complete resection are indispensable.

## Author Contributions

Takuma Ikeda contributed to patient management, data collection, and drafted the initial manuscript. Hirotaka Matsumoto, Shigenari Iwagaki, Ryo Ogawa, Yushi Shimamura, Emiko Saito, Takehisa Fukada, and Hiroaki Sakai supervised clinical care, contributed to the conception of the work, and critically revised the manuscript. All authors reviewed the final version of this manuscript and approved it to be published.

## Funding

The authors have nothing to report.

## Consent

The authors declare that written informed consent was obtained for the publication of this manuscript and accompanying images using the consent form provided by the Journal.

## Conflicts of Interest

The authors declare no conflicts of interest.

## Data Availability

The data that support the findings of this study are available from the corresponding author upon reasonable request.
